# Peutz-Jeghers syndrome: an unusual autopsy finding in pregnancy

**DOI:** 10.4322/acr.2021.279

**Published:** 2021-04-23

**Authors:** Michell Frank Alves de Oliveira, Maria Aparecida Marchesan Rodrigues

**Affiliations:** 1 Universidade Estadual Paulista (UNESP), Faculdade de Medicina de Botucatu, Departamento de Patologia, Botucatu, SP, Brasil

**Keywords:** Peutz-Jeghers Syndrome, Familial polyposis

## Abstract

Peutz-Jeghers syndrome (PJS) is a rare autosomal dominant polyposis entity that often remains undiagnosed. The major problems associated with PJS are acute complications due to (i) polyp-related intestinal obstruction, (ii) intussusception, and (iii) the risk of cancer in the long-term. We report the case of a 32-year-old female who presented at the emergency room with signs of acute abdomen and died during the clinical workup. She had a one-month history of nausea, vomiting, and diarrhea and was pregnant at about 30 weeks. There was no contributing past history except for undergoing small bowel resection in infancy. The postmortem examination revealed multiple arborizing polyps throughout the gastrointestinal tract, chiefly in the small bowel. Intestinal obstruction was found at the proximal jejunum with necrosis, perforation, and peritonitis. Histologically, the polyps were composed of tree branch-like bundles of smooth muscle covered by normal-appearing glandular epithelium, confirming the diagnosis of hamartomatous polyps. No malignant or premalignant lesions were detected in the gastrointestinal tract or other organs. This case was an opportunity to analyze the natural history and the pathological features of the Peutz-Jeghers syndrome in an adult and to investigate the presence of neoplastic lesions associated with this condition.

## INTRODUCTION

Peutz Jeghers syndrome (PJS) is a rare autosomal dominant syndrome that usually presents in childhood with multiple gastrointestinal hamartomatous polyps and mucocutaneous hyperpigmentation.^1^ ^,^ ^2^ It occurs with an estimated frequency of 1/50,000 to 1/200,000 individuals^3^ and is caused by a germline mutation of the serine-threonine kinase/ liver kinase B1 gene STK11 (formerly known as LKB1) on chromosome 19p13.^4^

The polyps are most common in the small intestine but may occur in the stomach and colon.^5^ ^-^ ^7^ PJS presents with a myriad of gastro- intestinal complications ranging from obstruction to intussusception, infarction, and bleeding, intussusception being the most frequently encountered in clinical practice.^8^ ^-^ ^11^ Intussusception is the most frequent gastrointestinal complication in PJS patients and starts early in childhood.^8^ ^-^ ^11^ Recurrent intestinal obstruction due to polyp size is seen in 43% of patients.^9^

Individuals with PJS have an increased risk for developing cancer in the gastrointestinal tract and at other sites.^12^ ^-^ ^14^ The lifetime risk for cancer in patients with PJS varies from 3% to 39%, but most gastrointestinal carcinomas do not develop from the hamartomatous polyps.^13^ ^-^ ^15^

In this study, we analyzed the pathological features of the Peutz-Jeghers syndrome in a young woman who developed a fatal complication during pregnancy due to upper gastrointestinal obstruction. The presence of associated pre-neoplastic and neoplastic lesions in the gastrointestinal tract and other sites was also investigated.

## CASE REPORT

A 32-year-old pregnant female at about the 30^th^ week of gestation presented at the emergency room complaining of nausea, vomiting, and diarrhea for one month. The colicky abdominal pain had intensified over the last three days. On the abdominal examination, upper abdominal and periumbilical tenderness was found. She had signs of acute abdomen and died during the examination at the emergency room. An antecedent of laparotomy at the age of 4 years was noticed, but no history of familial polyposis was available.

## AUTOPSY FINDINGS

**Figure 1:**
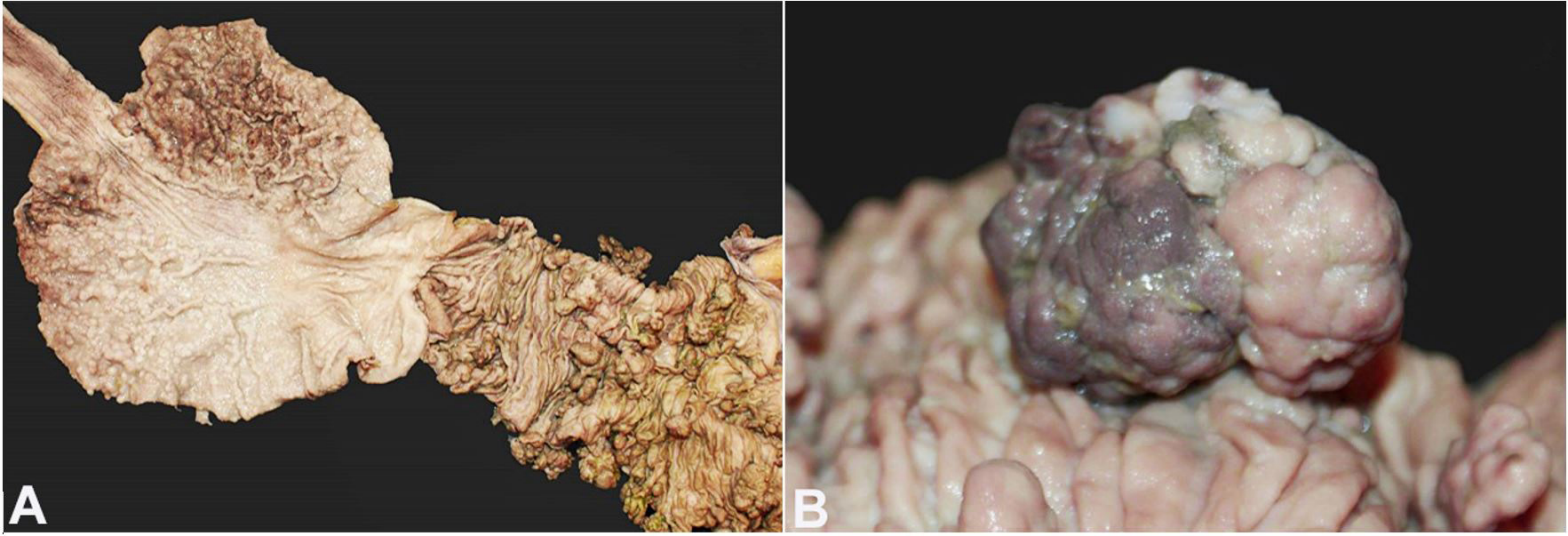
Gross findings of Peutz Jeghers syndrome. **A –** Duodenal dilatation with multiple arborizing polyps; **B –** Polyp necrosis in the proximal jejunum.

The postmortem examination revealed multiple arborizing polyps throughout the gastrointestinal tract with a predilection for the small bowel (Figure 1). The duodenum was dilated and covered by multiple polyps, pedunculated or sessile, of 0.1 to 4.0 cm (Figure 1A). Intestinal obstruction was found in the proximal jejunum, with polyp necrosis (Figure 1B), accompanied by perforation and peritonitis.

**Figure 2:**
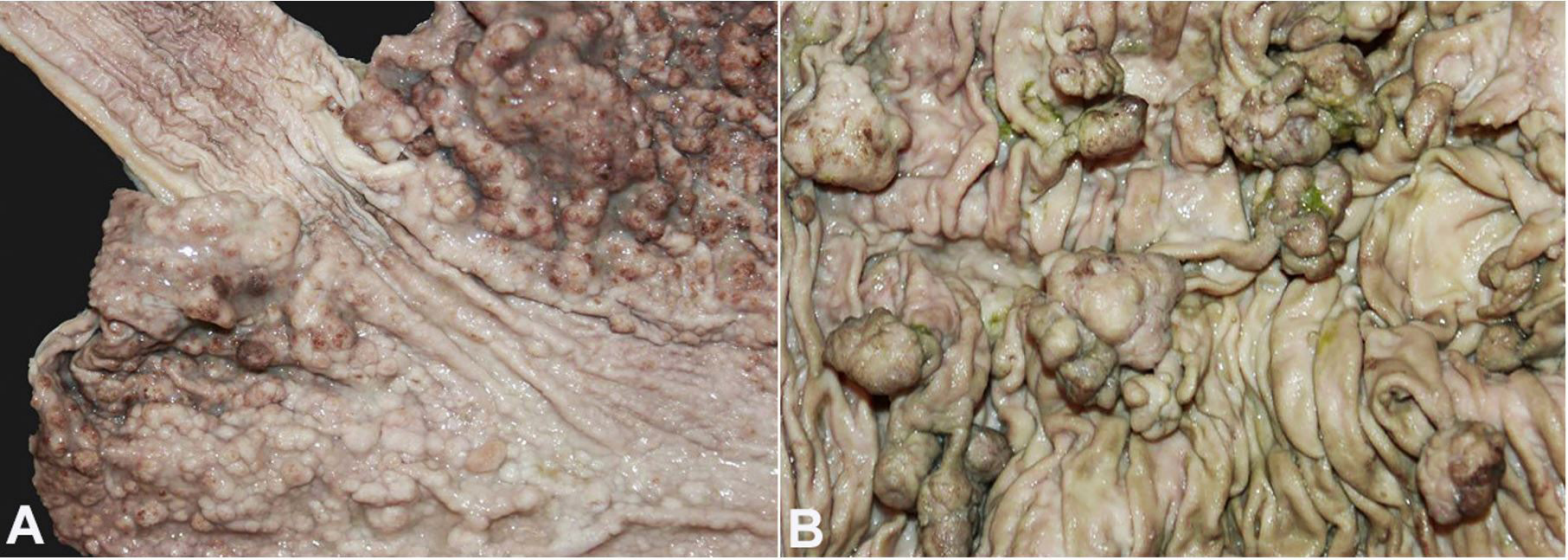
Gross features of Peutz Jeghers polyps. **A –** Small, sessile polyps in the stomach; **B –** Multiple arborizing polyps, pedunculated or sessile in the small intestinal mucosa.

In the stomach, there were multiple small, sessile polyps covering the gastric mucosa (Figure 2A). Figure 2B shows multiple arborizing polyps, pedunculated or sessile, covering the small intestinal mucosa.

**Figure 3:**
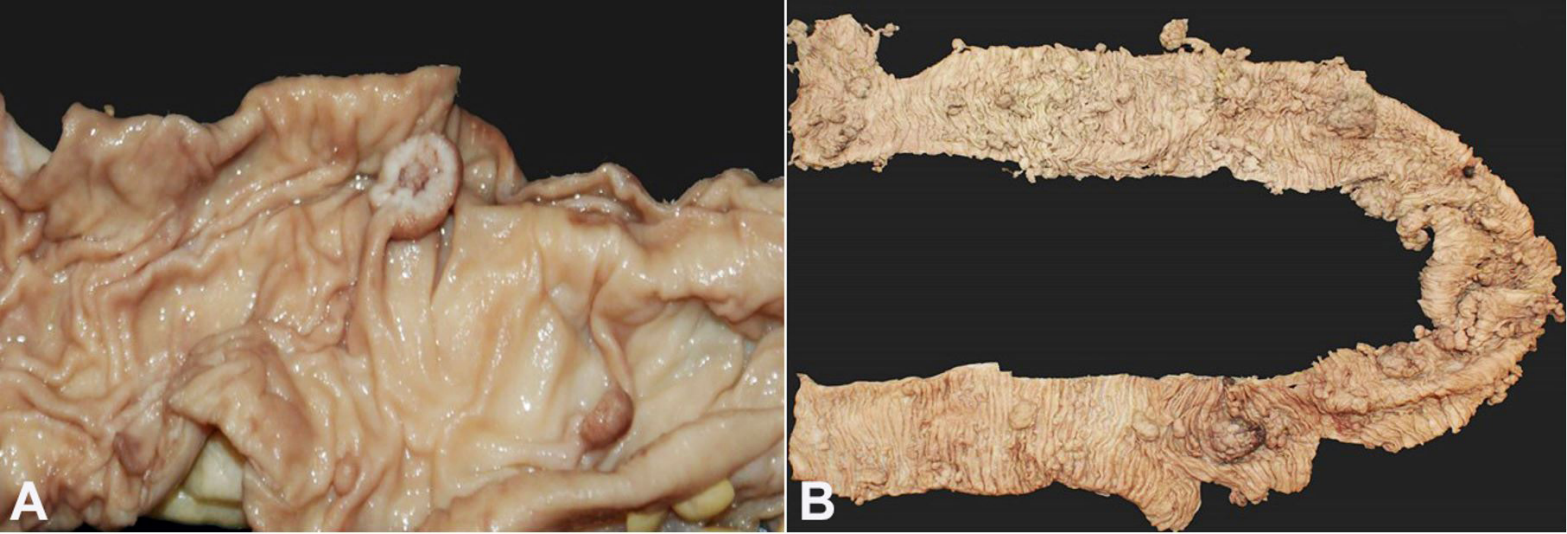
Gross features of Peutz Jeghers polyps in the colon. **A –** Few pedunculated polyps in the colonic mucosa; **B –** Large sessile masses throughout the colon.

In the colon, the polyps were fewer (Figure 3A), but some lesions were large sessile masses (Figure 3B). No polyps at extra-intestinal sites such as the bladder, bronchi, or gallbladder were found.

**Figure 4:**
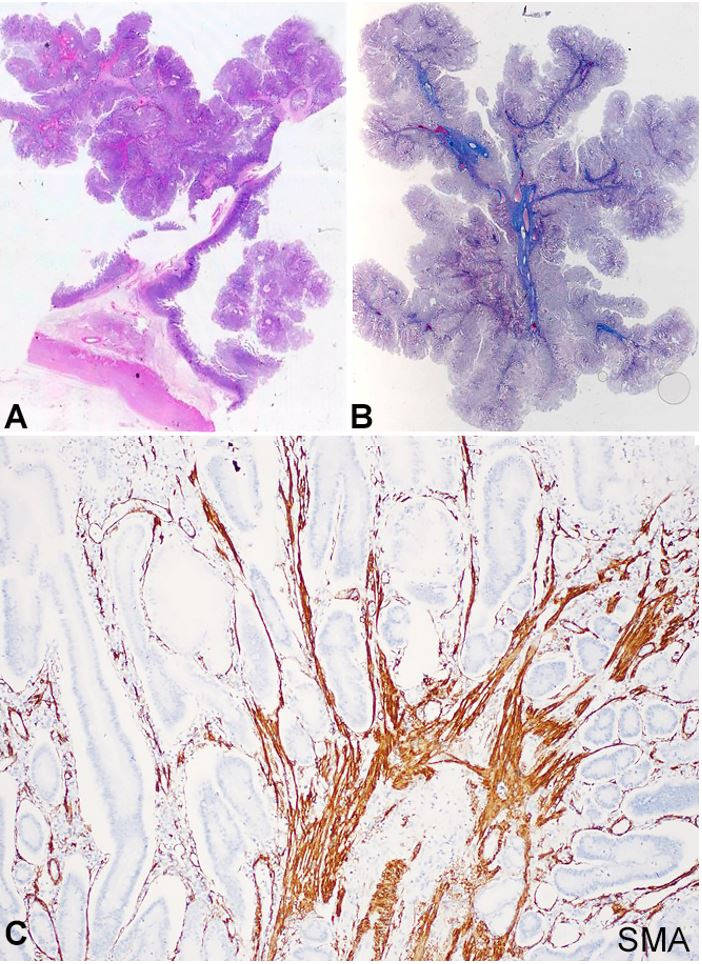
Histology of Peutz Jeghers polyps. Low power view demonstrates the arborizing pattern of organization in a small bowel polyp (**A**, H&E **B**) – Masson Trichrome; **C –** High power view shows arborizing smooth muscle bundles covered by mature small intestinal epithelium (SMA).

Histologically, all polyps were composed of tree branch-like bundles of smooth muscle covered by mature glandular epithelium (Figure 4A,B,C). This arborizing pattern of the organization was observed mainly in polyps from the small bowel. In the stomach and colon, the polyps did not show the distinctive arborization pattern of muscle fibers and looked similar to polyps associated with prolapse. There were no dysplasia nor adenomatous foci within the polyps throughout the gastrointestinal tract. No malignant lesions were detected in the gastrointestinal tract or in other organs. No mucocutaneous pigmentation was observed at post-mortem examination. Post-mortem data of the previous laparotomy revealed that she had a small bowel resection in infancy due to intussusception, with the pathologic finding of a hamartomatous polyp. However, she was not followed for monitoring further gastrointestinal complications.

## DISCUSSION

Peutz Jeghers syndrome (PJS) is a rare polyposis syndrome that still has many controversies. Although recognized as a clinical entity many years ago, our understanding of the pathologic features of the lesions and their potential relationship with cancer development is still not clear. In the present case, the diagnosis of PJS was made at postmortem examination due to a polyp-related upper intestinal obstruction. It was an opportunity to analyze the pathologic features of PJS in an adult patient in the fourth decade, as well as to investigate the presence of premalignant or malignant lesions in the gastrointestinal tract and other organs.

In the present case, the majority of the polyps were located in the small bowel, as observed in adult patients with PJS.^10^ ^,^ ^16^ In studies with pediatric patients, the distribution of gastrointestinal polyps is similar with 50% polyps in the small bowel, 36% in the stomach, and 21% in the colon.^6^ We have also observed polyps in the stomach and colon. Gastric lesions were small, sessile polyps grossly similar to other polyps. In the colon, the polyps were fewer, but some lesions were large sessile masses scattered throughout the colon. We did no find polyps at extra-intestinal sites such as the bladder, bronchi, or gallbladder.

Intussusception is the most frequent gastrointestinal complication in PJS patients and starts early in childhood.^8^ ^,^ ^9^ Approximately 95% of intussusceptions occur in the small bowel and are caused by hamartomas greater than >15mm.^9^ In our case, the patient had the first episode of small bowel intussusception in childhood at the age of 4 years, with the pathologic diagnosis of a hamartomatous polyp, but was not followed as a risk person for PJS. The post-mortem examination showed that the patient died of small bowel obstruction due to the polyps. The duodenum had a marked dilatation with multiple arborizing polyps covering the mucosa.

The clinical diagnosis of PJS is based on: (1) detection of three or more histologically confirmed Peutz-Jeghers polyps, or (2) any number of Peutz-Jeghers polyps with a family history of the syndrome, or (3) characteristic, prominent mucocutaneous pigmentation in a patient with a family history of the syndrome, or (4) any number of Peutz-Jeghers polyps in a patient with prominent mucocutaneous pigmentation.^16^ Therefore, the pathologic identification of the distinctive arborizing hamartomatous polyp is the hallmark for the diagnosis of Peutz-Jeghers syndrome.^16^ In our case, the arborizing pattern of smooth muscle fibers, covered by mature glandular epithelium, was observed mainly in polyps from the small bowel. Polyps of the stomach and colon did not show the distinctive arborization pattern of muscle fibers and looked similar to polyps associated with prolapse. One study on the morphologic features of colonic Peutz-Jeghers polyps showed that the arborizing pattern of smooth muscle proliferation occurred in 41% of polyps.^7^ In the stomach, the accuracy of distinguishing Peutz-Jeghers polyps from hyperplastic polyps and juvenile polyps was reported to be 18%.^17^ Therefore, the morphologic identification of a PJ polyp outside the small bowel may be challenging. The main differential diagnosis is with other hamartomatous polyps, particularly juvenile polyps.^7^ ^,^ ^17^

Mucocutaneous pigmentation is a hallmark feature for the diagnosis of PJS. Small, dark brown, oval, or circular macules occur most commonly on the lips, gums, oral mucosa, and hard palate.^11^ They are seen predominantly in infancy and may fade after puberty, as observed in the present case. Mucosal freckling is not pathognomonic for PJS since other conditions, including Carney complex and LEOPARD syndrome, are among the differential diagnoses.^18^ ^,^ ^19^

Another interesting aspect of the present case is that the patient was pregnant at the third trimester of gestation when the complication of PJS occurred. This might suggest that the polyp number and size would have increased during gestation due to hormonal stimulation and enhanced polyp-related complications such as intussusception. The influence of estrogen and progesterone on tumor growth during pregnancy has been suggested, but few reports on tumor growth at pregnancy are available.^20^ ^,^ ^21^ Well et al.^21^ analyzed the effect of pregnancy on the growth of neurofibromas in neurofibromatosis type 1 and did not find differences between pregnant and non-pregnant patients on plexiform growth and cutaneous neurofibromas.

The long-term cancer risk in PJS has been widely investigated. The majority of data on cancer risk in PJS comprises small single cohort studies.^11^ ^,^ ^14^ A difficult point to explain is how cancer arises in PJ polyp and the role of the PJS hamartomatous polyps in cancer development. PJS polyps are polyclonal, which is evidence against the malignant potential.^11^ Latchford et al.^15^ suggested that pancreatic and breast cancers are the most commonly seen cancers in PJS and that gastrointestinal cancer is of less clinical importance. In the present case, we did not find dysplasia or adenomatous foci within the hamartomatous polyps, and no malignant tumors were detected in the gastrointestinal tract or in other organs. Our findings add more data on the observation that most gastrointestinal carcinomas do not develop from hamartomatous lesions.^15^ ^,^ ^16^ Probably, cancer development in PJS may be related to a concomitant background of chromosomal instability.

In conclusion, this autopsy case report shows the natural history and pathological features of the Peutz-Jeghers syndrome in an adult woman who had a hamartomatous polyp in childhood that was not followed and developed a fatal complication during pregnancy due to upper intestinal obstruction.
